# Identification of *SAMD9* as an adaptive response gene to environmental changes and its association with overall survival and immunotherapeutic response in glioblastoma

**DOI:** 10.1186/s12935-025-04068-3

**Published:** 2025-12-02

**Authors:** Fan Li, Chang Lei, Min Luo, Weijian Bi, Yibo Li, Shihui Fu, Kaidi Yang

**Affiliations:** 1 Department of Pharmacy, Medical Security Center, Hainan Hospital of Chinese People’s Liberation Army General Hospital, Sanya, 572013 People’s Republic of China; 2 Department of Pharmacy, Sanya Rehabilitation and Recuperation Center of Joint Logistics Support Force, Sanya, 572000 Hainan People’s Republic of China; 3 Department of Urology, Jinling Hospital, Affiliated Hospital of Medical School, Nanjing University, 210015, Jiangsu Nanjing, People’s Republic of China; 4 Institute of Pathology and Southwest Cancer Center, Southwest Hospital, Third Military Medical University (Army Medical University), Chongqing, 400030 People’s Republic of China; 5 Department of Laboratory Pathology, Laoshan Medical District, 971 Hospital of PLA Navy, Qingdao, 266000 People’s Republic of China; 6 Department of Orthopedics, Central War Zone General Hospital, Wuhan, 430000 Hubei People’s Republic of China; 7 Department of Cardiology, Hainan Hospital of Chinese People’s Liberation Army General Hospital, Sanya, 572013 People’s Republic of China; 8 Department of Oncology, Hainan Hospital of Chinese People’s Liberation Army General Hospital, Sanya, 572013 People’s Republic of China

**Keywords:** *SAMD9*, IDH-wildtype glioblastoma, Immunotherapeutic resistance, Tumor microenvironment, Adaptive response gene, Molecular docking

## Abstract

**Background:**

Hypoxia and immune-suppressive microenvironments in glioblastoma drive transcriptional plasticity and phenotypic transition. Understanding these processes is crucial for overcoming therapy resistance and tumor relapse. This study investigates the expression pattern of sterile alpha motif domain-containing 9 (SAMD9) under these conditions, evaluates its prognostic value and spatial distribution, and explores its therapeutic implications.

**Methods:**

We analyzed SAMD9 expression and its prognostic value across three independent IDH-wildtype glioblastoma cohorts and validated findings via immunohistochemistry in human glioma tissues. We mapped its spatial distribution using the IvyGAP database and leveraged integrated single-cell and spatial transcriptomic data to delineate local cellular interactions. Drug sensitivities were predicted using the *oncoPredict* package, and molecular docking was performed with Autodock for drug screening. Key findings regarding *SAMD9* expression and its inducers were experimentally validated.

**Results:**

*SAMD9* was prominently expressed in an immune-suppressive subset of glioma tumor cells and was strongly associated with an interferon (IFN) signature. Elevated levels of *SAMD9* correlated with reduced efficacy of anti-PD-1 treatment. Spatial mapping revealed that SAMD9 was predominantly distributed in regions of microvessel proliferation and peri-necrotic niches, where SAMD9-positive tumor cells actively interacted with vascular cells and tumor-associated macrophages (TAMs). Hypoxia and TAM co-culture significantly upregulated SAMD9, suggesting a mechanism for Bevacizumab resistance. SAMD9-high tumors exhibited TAM-dominated immune infiltration, confirmed by immune signatures profiling and histological staining. Drug prediction and molecular docking identified the multi-kinase inhibitor Dasatinib as a promising therapeutic agent for SAMD9-high IDH-wildtype glioblastoma.

**Conclusions:**

*SAMD9* emerges as an adaptive response gene to environmental changes, exhibiting a significant immunomodulatory function, which highlights its promise as a therapeutic target for IDH-wildtype glioblastoma.

**Supplementary Information:**

The online version contains supplementary material available at 10.1186/s12935-025-04068-3.

## Introduction

Glioblastoma, IDH-wildtype, remains a formidable therapeutic challenge in neuro-oncology due to its inherent treatment resistance [1]. While the current standard of care relies on Temozolomide (TMZ)-based radio-chemotherapy, targeted therapies like Bevacizumab can extend progression-free survival, their efficacy is ultimately limited by rapidly acquired resistance and adverse side effects [2]. The promise of immunotherapy has also been undermined by the profoundly immunosuppressive tumor microenvironment (TME) of IDH-wildtype glioblastoma, which is characterized by a dominant myeloid cell compartment, a paucity of tumor-infiltrating lymphocytes (TILs), and signs of T cell exhaustion [3]. Thus, identifying specific immune-regulatory molecules capable of converting immunologically “cold” tumors into “hot” ones is crucial for developing effective therapeutic strategies.

This immunosuppressive TME is shaped by profound extracellular stresses, with hypoxia and immune pressure being key drivers of transcriptional plasticity and phenotypic transitions that fuel intra-tumoral heterogeneity [4, 5]. To adapt and survive, tumor cells employ additional strategies to remodel their environment [6, 7]. A classic example is the ability of hypoxic cells to orchestrate hallmark histological features like pseudopalisading necrosis and microvascular hyperplasia, which are linked to accelerated tumor growth and therapeutic failure [8]. Identifying molecules or genetic changes that enable tumor cell adaptation to these specialized niches is paramount for developing highly efficient targeted therapies.

The Sterile Alpha Motif Domain Containing 9 (*SAMD9*) gene on chromosome 7q21 encodes a protein involved in innate immune defense against infections, inflammatory responses, and antineoplastic activities [9–11]. It functions as a downstream target of TNF-α and interferon-γ (IFN-γ) signaling [12, 13]. While *SAMD9* has been characterized as a tumor suppressor in lung, breast, and colon cancer [14, 15], its role in glioma is paradoxical, as it has been reported to drive tumor progression by regulating HOXB8 [16]. SAMD9 is also under investigation as a potential antigen for mRNA vaccine development [17], and in low-grade glioma, it correlates with M2-polarized macrophage enrichment, contributing to tumor malignancy [18]. Yet, the precise regulatory mechanisms of SAMD9 and its impact on tumor immunity in diffuse gliomas, particularly IDH-wildtype glioblastoma, remain elusive.

The role of IFN-γ signaling in this context is similarly complex. Contrary to its canonical role in anti-tumor immunity, recent evidence suggests that a heightened tumor-intrinsic IFN response can predict adverse outcomes following immune checkpoint inhibition (ICI), potentially fostering immune tolerance and metastasis [19, 20]. Our previous findings revealed that a distinct glioma cell subset characterized by overexpression of IFN-γ responsive genes exhibited an immune-suppressive, ICI-resistant phenotype mediated by tumor-associated macrophage (TAMs) infiltration [21]. Unraveling the regulation and function of key molecules within this IFN-responsive axis is crucial for developing strategies to overcome immune evasion.

Given SAMD9’s role in antivirus and antitumor responses, and its potential as an IFN-response gene, we sought to comprehensively investigate its expression pattern and clinicopathological significance in IDH-wildtype glioblastoma. Through integrated analyses incorporating bulk, single-cell RNA-seq, and spatial transcriptome data, we demonstrate that SAMD9 is a hypoxia- and TAM-induced IFN-response gene strongly associated with an immunosuppressive TME. SAMD9-positive tumor cells localize to peri-necrotic and hypervascular niches, actively engaging with TAMs and vascular cells, and their expression predicts poor survival and resistance to anti-angiogenic and anti-PD-1 therapies. Our findings nominate SAMD9 as a promising prognostic biomarker and a potential therapeutic target for IDH-wildtype glioblastoma.

## Method and material

### Patient cohorts

Data and corresponding clinical information from The Cancer Genome Atlas (TCGA), Chinese Glioma Genome Atlas (CGGA), and Gravendeel datasets were obtained from the Gliovis data portal [22]. RNA-seq data (n = 154) and U133-Array data (n = 529) from TCGA were also retrieved from the TCGA official data portal. Expression profiles were correlated with clinical information, including histology, grade, IDH status, Verhaak’s molecular subtypes, and survival time. In accordance with the WHO 2021 classification, only adult-type diffuse astrocytic tumors with wild-type IDH status were classified as Glioblastoma, IDH-wildtype, and included in the primary study cohort [23]. RNA-seq data from normal brain tissue were acquired from the Genotype-Tissue Expression Project (GTEx) [24] for comparison with TCGA-GBM data after batch-effect correction using the *ComBat* algorithm from the *sva* R package. To evaluate the predictive value of *SAMD9* expression in treated glioblastomas, expression profiles from patients receiving Bevacizumab or anti-PD-1 therapy were downloaded from the Gene Expression Omnibus (GEO) with accession numbers GSE84010 [25] and GSE121810 [26]. Patients were classified as Bevacizumab-resistant if overall survival (OS) was < 1 year and Bevacizumab-sensitive if OS exceeded 3 years. Proteomic data for SAMD9 in normal and primary tumor tissues were obtained from the UALCAN portal [27] using the Clinical Proteomic Tumor Analysis Consortium (CPTAC) dataset.

### Tissue microarray

Glioma patients and tissue sample information for a glioma tissue microarray (TMA, n = 180, HbraG180Su02) were purchased from Shanghai Outdo Biotech Co. Ltd. Paraffin-embedded tumor tissues were employed for immunohistochemistry (IHC) staining. All TMA studies were conducted in accordance with the Declaration of Helsinki and were approved by the Ethics Committee of Shanghai Outdo Biotech Co. Ltd.

### Cell culture

The THP-1 cell line (human monocytic leukemia) and LN229 cell line (human glioblastoma) from the American Type Culture Collection (ATCC, Manassas, VA) were cultured in Dulbecco’s Modified Eagle Medium (DMEM, Gibco, C11995500BT) supplemented with 10% fetal bovine serum (FBS) (Gibco, A31608-02) and 1% penicillin/streptomycin (Beyotime, C0222). To establish a hypoxia model, LN229 cells were cultured under conditions of 1% O_2_, 94% N_2_, and 5% CO_2_ in an oxygen-adjustable incubator or incubated with cobalt chloride (CoCl_2_) at various concentrations and durations. THP-1 cells were polarized into a macrophage-like phenotype by stimulation with 100 nM PMA (Sigma-Aldrich, Shanghai, China) for 24 h. TAM differentiation was achieved by further incubation with 25 ng/mL IL-4 and 25 ng/mL IL-13. For co-culture experiments, TAMs or untreated THP-1 cells (1 × 10^5^) were seeded in 0.4 μm pore Transwell hanging inserts (Corning, USA) to assess their paracrine effects on tumor cells. To block specific cytokines, TAM-conditioned medium was incubated with neutralizing antibodies (all from Invitrogen) against TNF-α (10 µg/mL), IFN-γ (10 µg/mL), TGF-β (5 µg/mL), IL-6 (1 µg/mL), and IL-10 (5 µg/mL).

### Immune-related signature

The TME contexture was characterized using the *IOBR* R package, which integrated eight established methodologies to calculate Single-sample Gene Set Enrichment Analysis (ssGSEA) scores [28]. TME signature scores, derived from mRNA profiles, were calculated using the *iobr_cor_plot* function for comparisons between *SAMD9*^low^ and *SAMD9*^high^ groups. Additionally, the *CIBERSORT* [29] and *xCell* [30] algorithms*,* implemented within *IOBR,* were used for deconvolution analysis to estimate the absolute abundance of immune and stromal compartments.

### Genomic alteration analysis

Somatic genomic alterations of *SAMD9* were summarized, analyzed, and visualized using the cBioPortal platform (http://www.cbioportal.org/). Co-occurrence and mutual exclusivity between *SAMD9* and other major glioma-driven mutations were assessed using the Mutual Exclusivity module in cBioPortal and visulized with an oncoplot.

### Integrated single-cell and spatial transcriptomic analyses

A Glioblastoma, IDH-wildtype scRNA-seq dataset was retrieved from Sequence Read Archive (SRA, PRJNA579593) and processed as previously described [21]. Malignant cells were distinguished fromnon-malignant cells using the *inferCNV* algorithm. The annotated single-cell object served as a reference for spatial transcriptomic data integration. Publicly available spatial gene expression datasets for Glioblastoma, IDH-wildtype, were sourced from 10 × Genomics Platform and Ravi’s repository (Datadryad) [5] (see Table S1 for details). Data were processed using Space Ranger 1.2.0, and subsequent analysis was performed in R (v4.0.4) using the *Seurat* package (v4.1.0). Spots were filtered based on the following thresholds, including detected gene count below 200, detected UMI below 1000, and mitochondrial percent over 25%. Genes detected in fewer than three spots were removed. The spot matrix was normalized using the *SCTransform* function to eliminate confounding sources of variation. Cell type labels were transferred from the scRNA-seq reference to the spatial data using the *FindTransferAnchors* function. The spatial distribution of cell subsets was visualized with the *SpatialFeaturePlot* function. To quantify cellular interactions, the Euclidean distances between SAMD9-positive  tumor cell subset TC-6 (and other subsets) and the nearest TAM-1/2 or vascular cells were calculated and compared to distances from randomly selected spot groups of equivalent size.

### Spatial exploration of gene expression patterns through IvyGAP

Cellular-resolution colorimetric in situ hybridization (ISH) data for SAMD9 were retrieved from the Ivy Glioblastoma Atlas Project (IvyGAP) data portal. The spatial expression pattern of SAMD9 across anatomically distinct regions of gliomas was analyzed using IvyGAP’s online visualization and analysis tools.

### Enrichment analysis

Gene-set enrichment analysis (GSEA) was performed to identify Hallmark and Reactome gene sets (from the MSigDB database) enriched in *SAMD9*^High^ samples compared with *SAMD*9^Low^ samples. GSEA results were visualized using the *gseaplot2* function within the *enrichplot* package. Enriched terms were illustrated using the *Dotplot* function, sorted by the normalized enrichment score (NES).

### Immunoblot assay

For Western blotting, 5 × 10^6^ cells were harvested for protein extraction using RIPA cell lysis reagent (ThermoFisher, 89900, USA). Proteins were denatured, and 30 μg per lane was loaded onto 10% SDS-PAGE gels and transferred to PVDF (polyvinylidene difluoride) membranes. The membranes were then incubated overnight at 4 °C with primary antibodies against SAMD9, β-actin, and HIF-1α, followed by incubation with an HRP-conjugated secondary antibody (Cell Signaling Technology). Protein bands were detected using an ECL kit (Advansta, K-12045-D10, USA) and imaged with a ChemiDoc XRS Imaging System (Bio-Rad).

### Quantitative real-time PCR

Quantitative real-time PCR assay was performed as detailed previously [31], using the following primers for *SAMD9*:

*SAMD9*-F:5′-ATGGCAAAGCAACTTAACCTTCC-3′;

*SAMD9*-R:5′-CCATTCACGTCTTGTTCAGTCA-3′.

For *β-Actin*:

*β-Acin*-F:5′-CATGTACGTTGCTATCCAGGC-3′;

*β-Acin*-R:5′-CTCCTTAATGTCACGCACGAT-3′.

### Immunohistochemistry 

IHC staining was performed on formalin-fixed, paraffin-embedded glioblastoma sections following standard protocols. Two neuropathologists, blinded to the *SAMD9* status, independently assessed the staining. A semi-quantitative scoring system based on staining intensity and the percentage of positive tumor cells was applied, as previously described [21]. Representative IHC staining images for different glioma grades were obtained from The Human Protein Atlas. Antibodies used are listed in Table S2.

### Drug prediction and molecular docking

The *oncoPredict* R package [32] was used to predict the sensitivity of *SAMD*9^hig^^h^ IDH-wildtype glioblastoma samples to 365 compounds from the Genomics of Drug Sensitivity in Cancer (GDSC) database, which integrates extensive drug sensitivity and genomic datasets to identify new therapeutic biomarkers [33]. The calculated half-maximal inhibitory concentration (IC50) for each compound was correlated with *SAMD9* expression. Candidate drugs were selected based on a correlation index > 0.2 and log2fold-change[mean(*SAMD*9^low^ IC50)/mean(*SAMD*9^high^ IC50)] > 0.8. The Autodock tool was employed to predict the binding of these drug candidates to the SAMD9 protein structure from the Protein Data Bank (PDB) archive. The small molecule structures of drugs were obtained from the PubChem Database, and molecular docking was performed according to standard protocols. High and stable binding affinity between ligands and biological macromolecules was determined based on the lowest binding energy. Results were visualized using PyMol (v2.6, Open-Source). Drug effects on the *SAMD9*-correlated signature were assessed using microarray data with accession numbers GSE134432, GSE164437, and GSE74557.

### Statistical analysis and data visualization

A volcano plot for differential expressed genes (DEGs) analysis was generated using the *ggplot* package. Correlation analyses were performed using Pearson’s correlation coefficient and visualized with the *ggstatsplot* function from the *ggstatsplot* R package. Kaplan–Meier survival curve were constructed to compare survival differences, with log-rank tests used for comparisons. The median expression of SAMD9 was used as the cutoff to define high and low groups. Continuous variables were compared using the unpaired two-tailed Student’s *t*-test (for normal distribution) or the Wilcoxon rank-sum test (for non-normal distribution). A *p*-value < 0.05 was considered statistically significant. All statistical analyses were performed using Graphpad Prism 8.0 and R software (version 4.0.4).

## Results

### SAMD9 exhibited prominent expression in immunosuppressive tumor cell subset

**Fig. 1:**
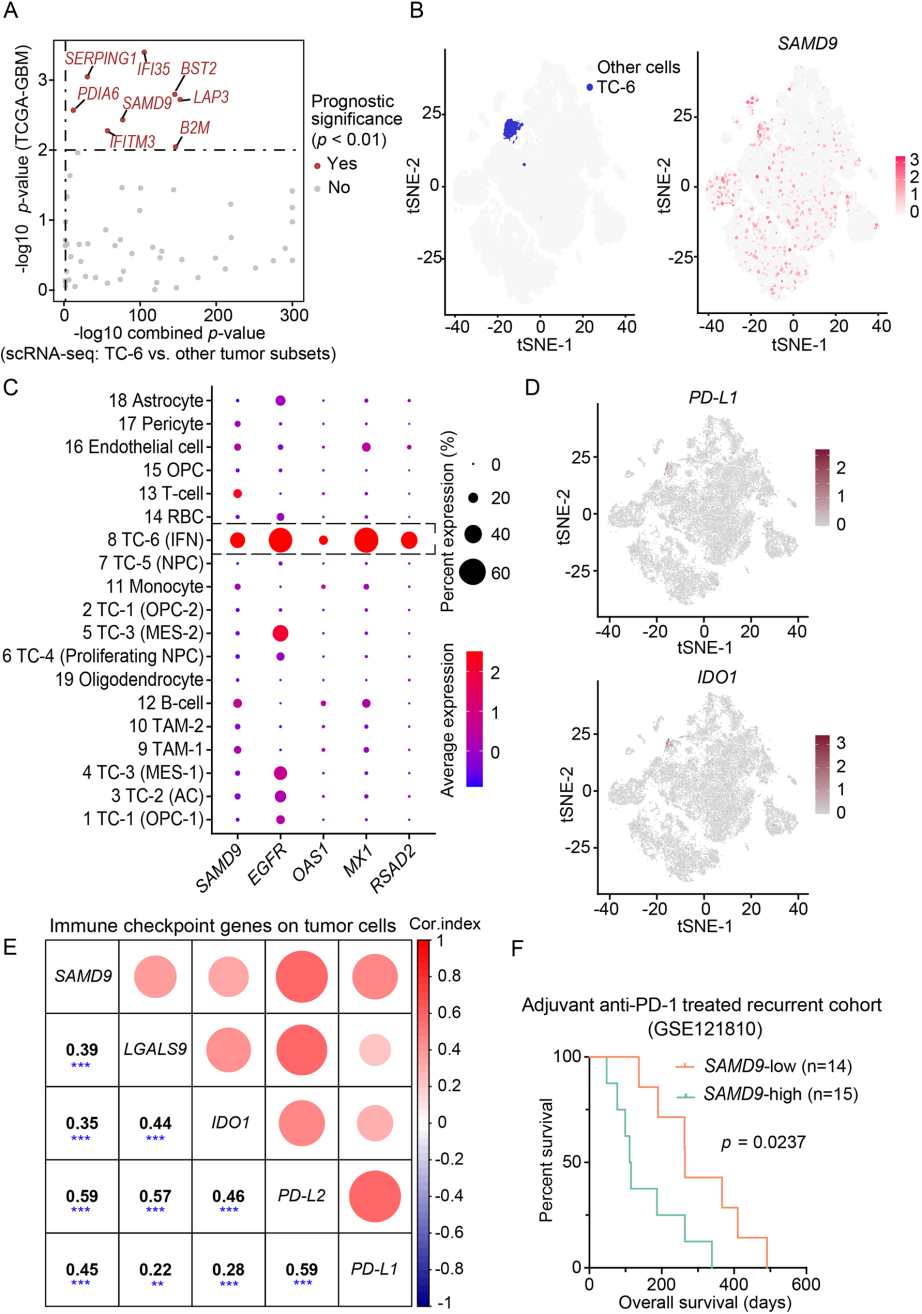
*SAMD9* exhibited prominent expression in immune-suppressive tumor cell subset. A Scatter plot of 64 significantly differentially expressed genes (DEGs) in TC-6 compared to other tumor subsets. The x-axis represents the combined *p*-value from scRNA-seq differential expression analysis. The y-axis indicates the -log10(*p*-value) for survival association in the TCGA cohort. Red dots highlight genes with significant prognostic value (*p* < 0.01). B The right panel displays a t-SNE plot depicting *SAMD9* expression, the matched left panel highlights the annotated TC-6 subset in blue. C Dot plots illustrating the expressions of *SAMD9* and interferon-stimulated genes (ISGs) across all cell subsets. TAM, Tumor-associated macrophages; RBC, Red blood cell; OPC, Oligodendrocyte progenitor cell; NPC, Neural progenitor cell; AC, Astrocyte; MES, Mesenchymal; IFN, Interferon. D t-SNE plots showing the expression patterns of immune checkpoint genes (*IDO1* and *PD-L1*). E Correlation analysis between *SAMD9* and cancer cell-expressed immune checkpoint genes. In the upper triangle, circle size and color represent the correlation strength, while the lower triangle displays Pearson correlation coefficients. ***, *p* < 0.001; **, *p* < 0.01. F Kaplan–Meier analyses of overall survival in recurrent glioblastoma patients treated with anti-PD-1 therapy, stratified by median *SAMD9* expression

Our previous investigation [21] constructed a single-cell atlas of five human Glioblastoma, IDH-wildtype tumors, which identified a distinct tumor cell subset termed tumor cell (TC)-6. This subset exhibited features consistent with an immune-suppressive phenotype (Fig. S1A). Further analysis identified 64 conserved markers in TC-6 across multiple scRNA-seq samples. Among these, *SAMD9* was significantly associated with poor patient prognosis, establishing its clinical relevance (Fig. 1a). Notably, *SAMD9* expression was highest in the TC-6 subset and showed a strong positive correlation with interferon-stimulated genes (ISGs), including *OAS1*, *MX1*, and *RSAD2* (Fig. 1b, c). TC-6 displayed an immune-exclusive phenotype, characterized by the expression of immune checkpoint genes (Fig. 1d). This role in immune modulation was further supported by a significant correlation between *SAMD9* and canonical immune checkpoint molecules in the TCGA bulk tissue transcriptome, suggesting its potential role in tumor immune escape (Fig. 1e and Fig. S1B). Critically, in a cohort of recurrent glioblastoma patients receiving adjuvant anti-PD-1 therapy, high *SAMD9* expression predicted worse clinical outcomes (Fig. 1f). Collectively, these findings prompted further investigation into the molecular characteristics and functions of SAMD9.

### SAMD9 expression levels in relation to clinicopathological parameters and patient survival

**Fig. 2:**
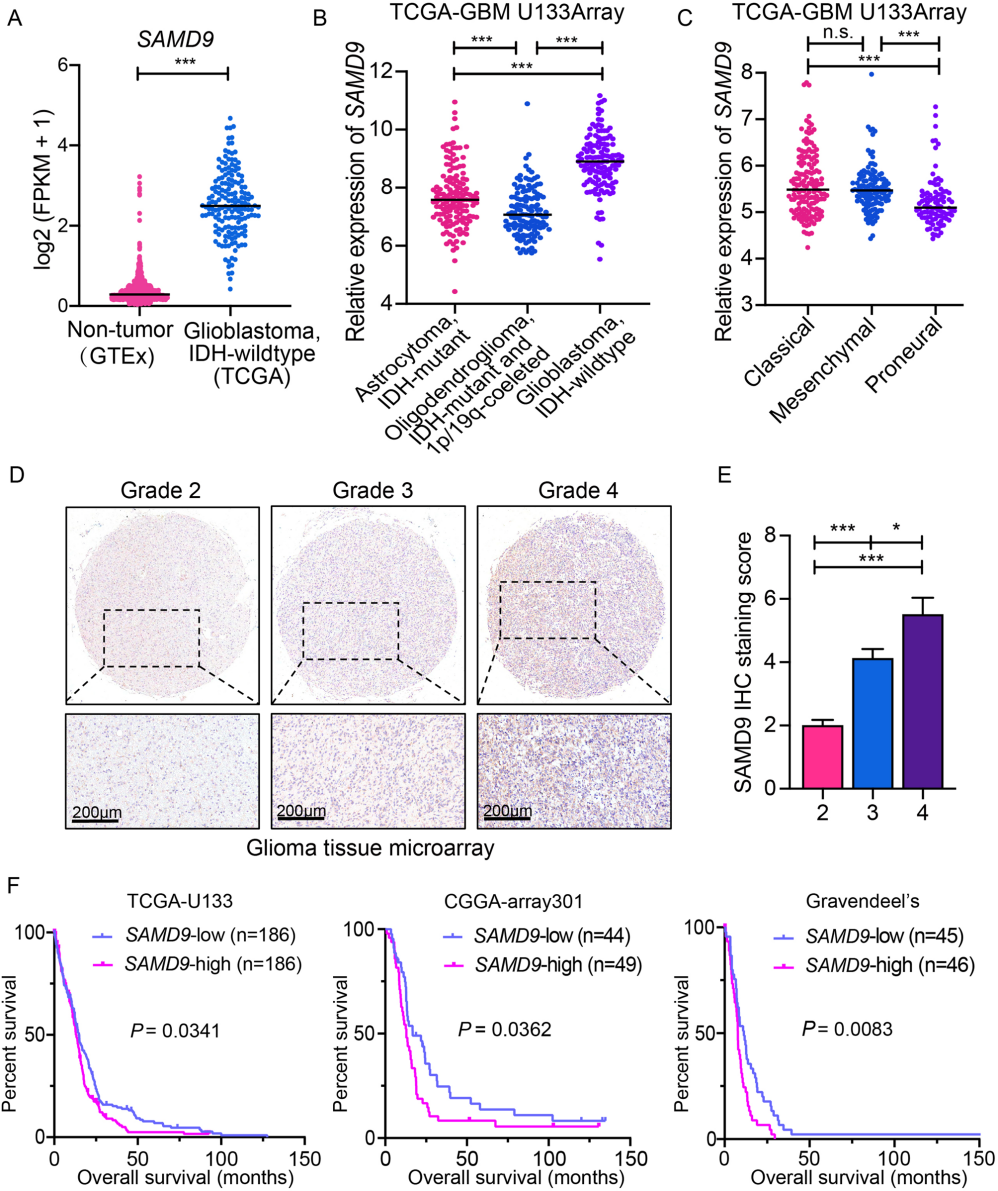
*SAMD9* expression levels relating to clinicopathological parameters and patient survival. A *SAMD9* mRNA expression in a combined RNA-seq dataset from TCGA (glioblastoma, IDH-wildtype) and GTEx (normal brain). Data are presented as log2-transformed FPKM. B *SAMD9* mRNA expression across glioma histological subtypes. C *SAMD9* mRNA expression in IDH-wildtype glioblastoma stratified by molecular subtype. D Representative immunohistochemistry (IHC) staining of SAMD9 in a glioma tissue microarray across WHO grades 2–4. Scale bar = 200 μm. E Quantification of SAMD9 IHC staining intensity across glioma grades. Data are presented as mean ± SEM. ***, *p* < 0.001; *, *p* < 0.05. F Kaplan-Meier overall survival analysis of patients with IDH-wildtype glioblastoma from the TCGA-U133 (left), CGGA (middle), and Gravendeel (right) cohorts, stratified by *SAMD9* expression

We further investigated the clinical relevance of *SAMD9* expression patterns in glioblastoma. *SAMD9* mRNA expression was significantly upregulated in IDH-wildtype glioblastoma compared to normal brain tissue, as evidenced by TCGA and GTEx data (Figs. 2a and S2A).This upregulation was inversely correlated with reduced promoter methylation (Fig. S2B). Stratification of gliomas according to the latest WHO 2021 classification revealed elevated *SMAD9* mRNA levels in IDH-wildtype glioblastoma compared to both IDH-mutant astrocytomas and oligodendrogliomas (Fig. 2b). Analysis of TCGA data across molecular subtypes revealed predominant *SAMD9* expression in the Mesenchymal and Classical subtypes (Fig. 2c). Furthermore, low *SAMD9* expression was correlated with Glioma CpG island methylator phenotype (G-CIMP) (Fig. S2C), and significant differences were observed between primary and secondary tumors (Fig. S2D).

IHC staining images from the Human Protein Atlas and proteomic data from the UALCAN portal confirmed SAMD9 protein overexpression in glioblastoma relative to normal brain and low-grade gliomas (Fig. S2E-F). Subsequent IHC analysis on a paraffin-embedded glioma tissue TMA revealed a clear grade-dependent increase in SAMD9 protein levels (Fig. 2d). Quantification analysis confirmed a positive correlation between SAMD9 expression and malignancy grade (Fig. 2e). Furthermore, the prognostic value of *SAMD9* was evaluated in three IDH-wildtype glioblastoma datasets (TCGA, CGGA, and Gravendeel). Consistently, *SAMD9*^high^ patients exhibited significantly worse survival compared to *SAMD9*^low^ patients (Fig. 2f). Multivariate Cox regression analysis identified SAMD9 as an independent prognostic factor after adjusting for key clinical parameters (Fig. S2G). Given the established link between genomic alterations and tumor immunity [34], we analyzed *SAMD9* genomic alteration features using the cBioPortal portal. Approximately 5% of glioblastoma cases showed *SAMD9* amplification or missense mutation. Co-occurrence analysis revealed that *EGFR was* the only gene with a significant concurrent alteration pattern (log2 odds ratio > 2, *p* = 0.002; Fig. S3A). As a frequently altered oncogene in up to 60% of cases [35], *EGFR* alteration was the most common co-occurring event in *SAMD9*-altered tumors. Additionally, alterations in *SEPTIN14*, *CALCR*, and *FAM133B* were significantly more prevalent in *SAMD9*-altered tumors relative to unaltered tumors (Fig. S3B).

### Spatial expression pattern of SAMD9

**Fig. 3:**
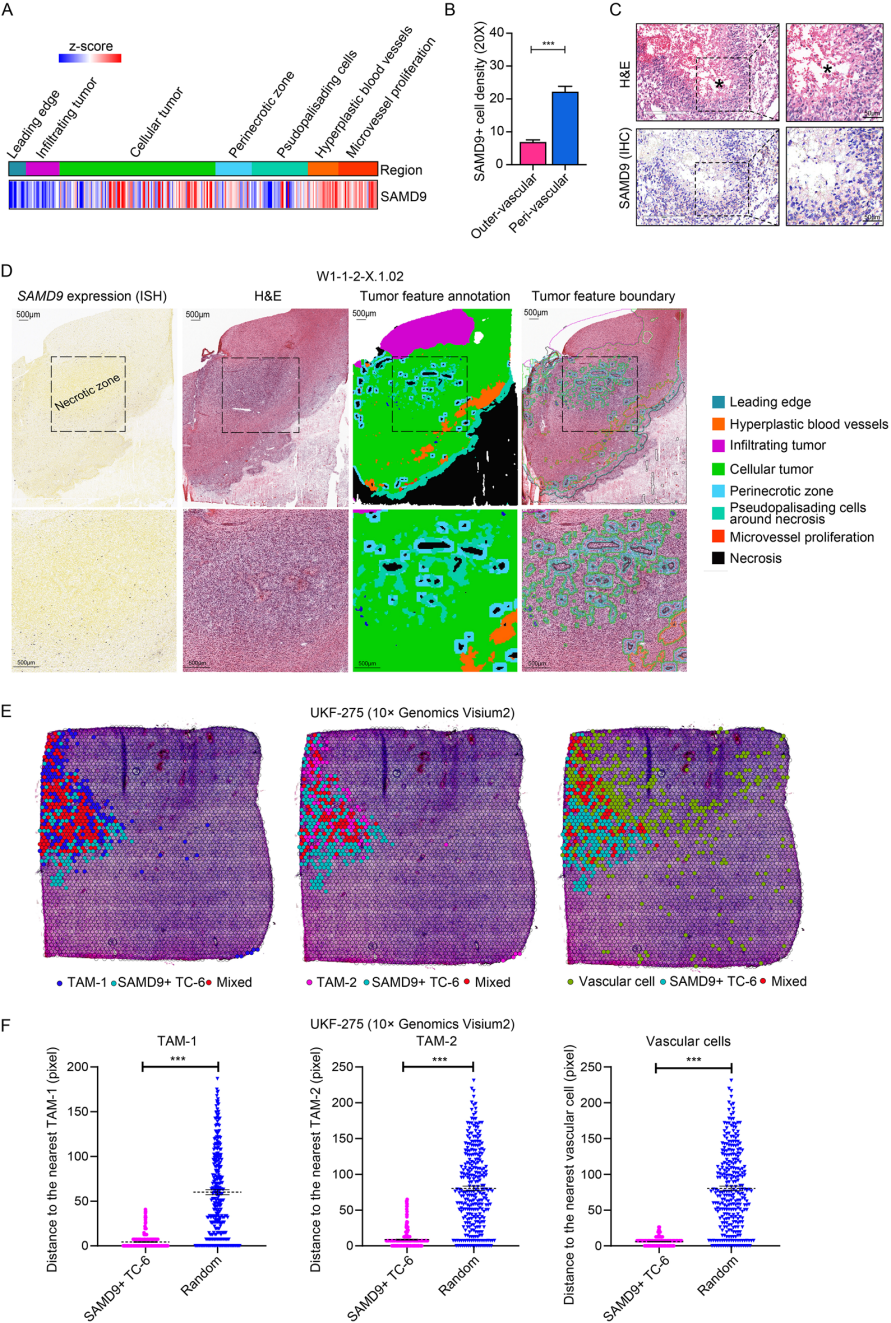
*Spatial expression pattern of SAMD9.* **A** Heatmap of *SAMD9* expression across glioblastoma samples from IvyGAP, categorized by histology region. **B** Quantification of SAMD9 IHC staining intensity in outer-vascular versus peri-vascular regions (defined as areas within 25 μm of microvessels). Data were presented as mean ± SEM. **C** Representative IHC staining of SAMD9 with matched H&E staining in human glioblastoma tissues. Boxed regions are shown as magnified insets. Necrotic areas are marked with asterisks (*). Scale bar = 500 μm. **D** Distribution pattern of *SAMD9* expression by in-situ hybridization (ISH) in human glioblastoma tissue. Panels show (left to right): ISH signal (black dot), H&E staining, tumor feature annotation, and tumor feature boundaries with color key. Scale bar = 500 μm. **E** Computational mapping of TAMs, vascular cells, and SAMD9-positve tumor subsets on Visium spatial transcriptomics data, with each cell type color-coded. **F** Scatter plots showing the distance from the SAMD9-positive  TC-6 subset and randomly selected control spots to the nearest TAM-1, TAM-2 and vascular cells. ***, *p* < 0.001

To delineate the spatial expression pattern of *SAMD9*, we leveraged the IvyGAP atlas, which integrates detailed anatomical, spatial molecular, and histological data [36]. This analysis revealed significant spatial transcriptomic heterogeneity of *SAMD9* across different anatomical regions, with prominent expression in the Microvascular Proliferation (MVP) region, as confirmed by IHC (Fig. 3a, b). Partial expression was observed in the Cellular Tumor and Perinecrotic Zone (Fig. 3a). Complementary in-situ hybridization (ISH) and IHC analyses further localized SAMD9 to niches surrounding necrotic regions (Fig. 3c, d). Advances in spatial transcriptomics, coupled with their derived integrated algorithms, enable the precise dissection of cell types within multi-cellular spatial spots [37]. Through a conjoint analysis of spatial gene-spot matrices, we discovered that SAMD9-positive tumor cells exhibited a closer proximity to TAMs (TAM-1 and TAM-2 subsets) and vascular cells than to randomly generated spots (Fig. 3e, f). In contrast, other tumor cell subsets did not present such niche patterns (Fig. S4C). Validation using an additional spatial transcriptiomics atlas corroborated that SAMD9-expressing tumor cells reside in niches characterized by intense intercellular interactions with TAMs and vascular cells (Fig. S4A-C).

### Increased SAMD9 expression under hypoxic conditions and TAM co-culture

**Fig. 4:**
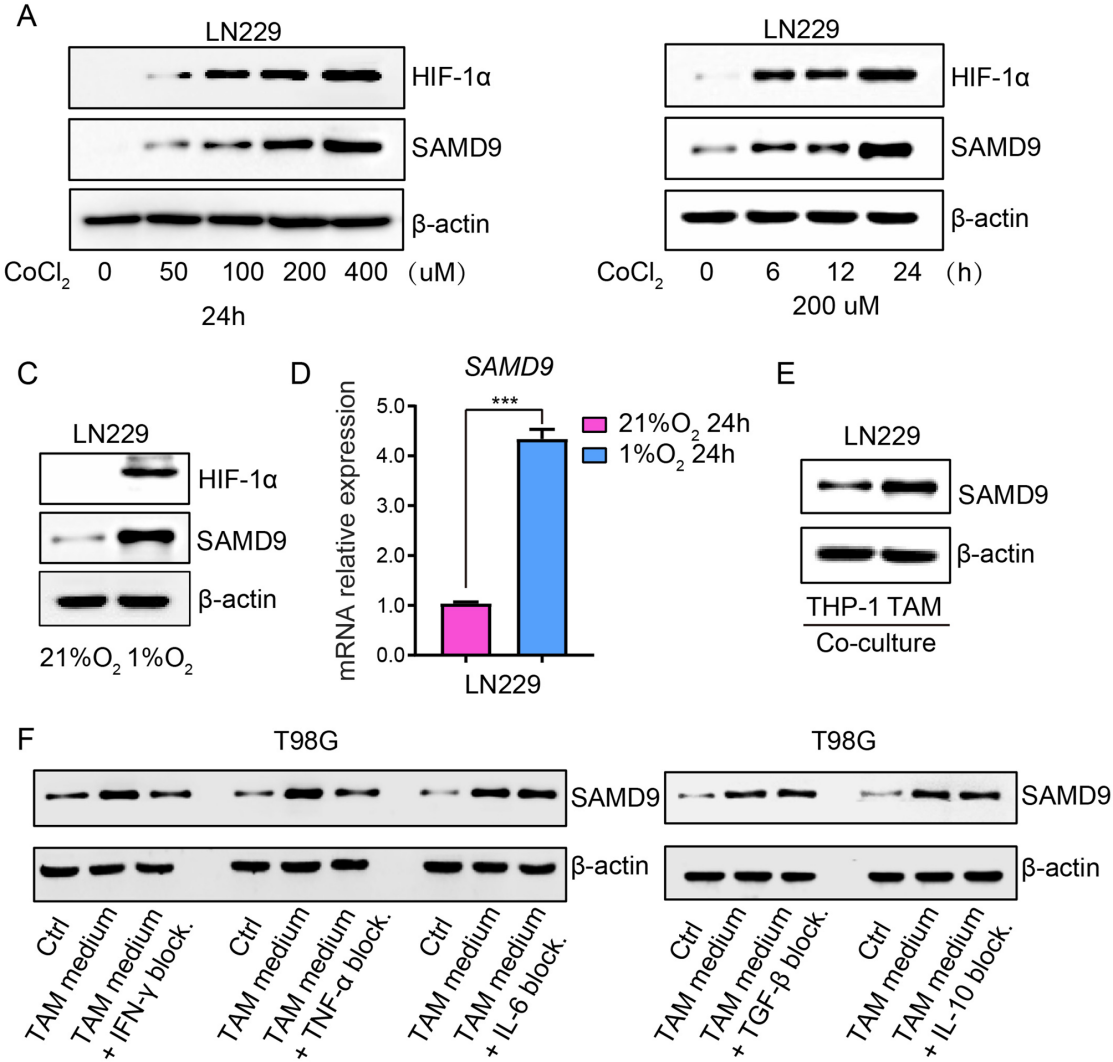
*Increased SAMD9 expression under hypoxic conditions and TAM co-culture.* **A** Western blot analysis of HIF-1α and SAMD9 protein levels in LN229 cells treated for 24 hours with increasing concentrations of cobalt chloride (CoCl_2_). β-actin served as a control. **B** Detection of HIF-1α, SAMD9, and β-actin by Western blot in LN229 cells was performed after treatment with CoCl_2_ at 200 μM at 0, 6, 12, and 24 hours. **C**, **D** SAMD9 expression in LN229 cells under hypoxic conditions (1% O_2_) for 24 h, assessed at the protein (C) and mRNA (D) levels. ***, *p* < 0.001. **E** SAMD9 protein expression in LN229 cells after 24-hour co-culture with untreated THP-1 monocytes or THP-1-derived TAMs. **F** SAMD9 expression levels in T98G cells treated for 24 hours with TAM-conditioned medium that was pre-incubated with or without neutralizing antibodies against the specific cytokines

Pseudopalisading cells surrounding necrotic regions in glioblastoma are characterized by HIF-1α accumulation and upregulation of hypoxia-inducible genes that drive angiogenesis, mesenchymal transition, and invasion [38, 39]. Given the high expression of SAMD9 in the Mesenchymal subtype and peri-necrotic region, we investigated its response to hypoxia. Treatment of LN229 glioblastoma cells with CoCl_2,_ a chemical hypoxia inducer that stabilizes HIF-1α/HIF-2α under normoxic conditions [40], resulted in a dose- and time-dependent increase in SAMD9 protein levels (Fig. 4a, b). This was further corroborated by the upregulation of SAMD9 at both transcriptional and protein levels under hypoxia conditions (1% O_2_) (Fig. 4c, d). Consistent results were observed in T98G glioblastoma cells (Fig. S5A-B). Considering the prominent infiltration of TAMs and their spatial association with SAMD9-positive tumor cells, we established a co-culture system. SAMD9 expression was significantly elevated in tumor cells co-cultured with THP-1-derived TAMs compared to those exposed to untreated THP-1 cells (Figs. 4e and S5C). Among key TAM-secreted factors, blocking TNF-α, IFN-γ in the TAM-conditional medium efficiently suppress SAMD9 upregulation (Fig. 4f). This suggests that *SAMD9* is a tumor microenvironment-responsive gene, induced by both hypoxic stress and TAM-derived inflammatory signals.

### SAMD9 expression predicts Bevacizumab resistance and informs candidate therapeutic strategies

**Fig. 5:**
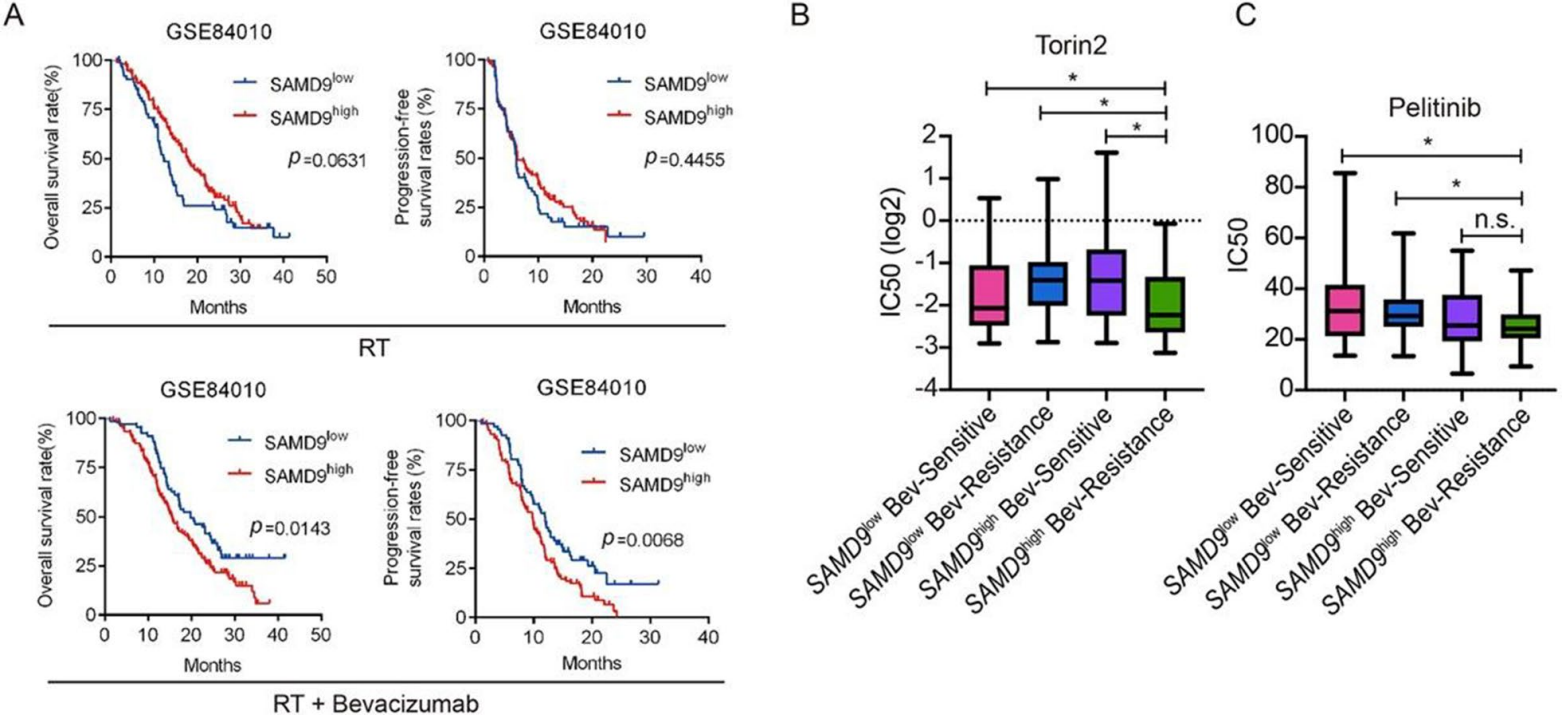
*SAMD9* expression predicts Bevacizumab resistance and informs candidate therapeutic strategies. **A** Overall survival and progression-free survival of glioblastoma patients receiving Radiotherapy & Temozolomide (RT) or RT plus Bevacizumab, stratified by *SAMD9* expression using a uniform cutoff. **B**, **C** Predicted semi-inhibition concentration (IC50) values for Torin2 (**B**) and Pelitinib (**C**) in glioblastoma patients stratified by *SAMD9* expression and Bevacizumab sensitivity. *, *p* < 0.05; n.s., not significant

Chronic treatment with Bevacizumab, while effective in reducing vascularity, frequently induces intratumoral hypoxia and enhances TAM infiltration, ultimately leading to therapeutic resistance in glioblastoma [41, 42]. To test the specific contribution of SAMD9 to this resistance, we stratified patients receiving standard Radiotherapy (RT) with or without Bevacizumab into *SAMD9*^high^ and *SAMD9*^low^ subgroups based on a uniform cutoff. High *SAMD9* expression predicted poor prognosis in patients treated with RT plus Bevacizumab (Fig. 5a and Table S3). Notably, this predictive value was absent in patients receiving RT alone (Fig. 5a), indicating a specific role for SAMD9 in Bevacizumab resistance.

To explore potential therapeutic agents for *SAMD9*^high^ resisitant glioblastoma, we utilized the *oncoPredict* algorithm and the GDSC database to estimate IC50 of 365 compounds. Stratifying patients by *SAMD9* expression and drug sensitivity identified Torin2, a potent mTOR inhibitor known to activate autophagy [43], as a candidate agent for *SAMD9*^high^-resistant tumors (Fig. 5b). Additionally, Pelitinib, an irreversible EGFR tyrosine kinase inhibitor currently in clinical trials for solid tumors, showed promise in reversing Bevacizumab resistance (Fig. 5c).

### SAMD9-high glioblastomas exhibit interferon-driven molecular features and an immunosuppressive microenvironment

**Fig. 6:**
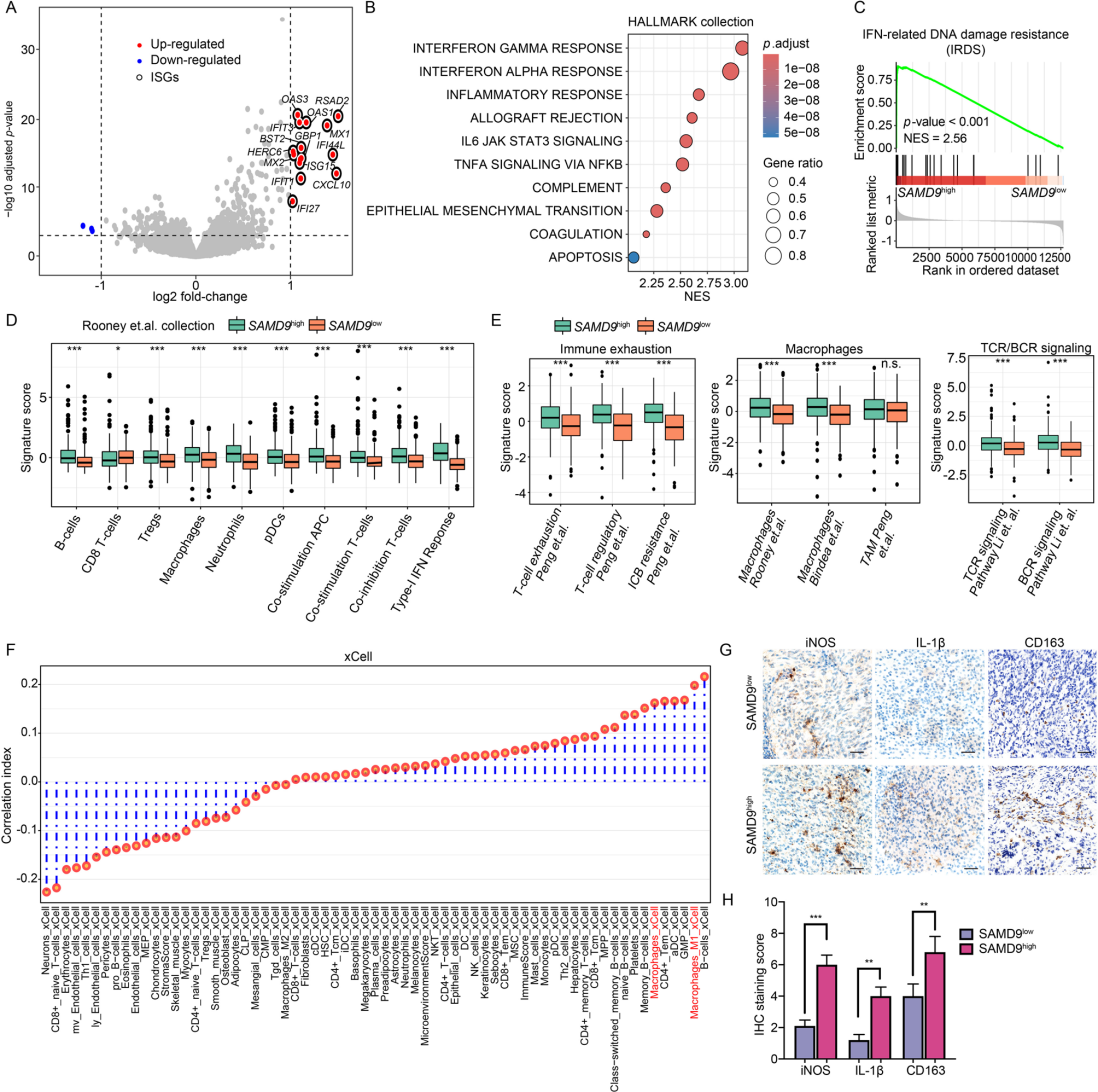
*SAMD9-high glioblastomas exhibit interferon-driven molecular features and an immunosuppressive microenvironment.* **A** Volcano plot of DEGs between *SAMD9*^high^ and *SAMD9*^low^ Glioblastoma, IDH-wildtype samples from TCGA (|fold-change|> 2, FDR < 0.05). Red and blue dots represent up- and down-regulated genes, respectively. Interferon-stimulated genes (ISGs) are circled in black. **B** Bar plot of Gene Set Enrichment Analysis (GSEA) results for Hallmark pathways in *SAMD9*^high^ versus *SAMD9*^low^ glioblastomas. Bar color and size corresponding to adjusted *p*-values and gene ratios, respectively. **C** GSEA plot demonstrating enrichment of the IFN-related DNA damage resistance signature (IRDS) in *SAMD9*^high^ glioblastomas. **D** Boxplots showing expression scores for immune-related terms from Rooney et.al. collection in *SAMD9*^high^ versus *SAMD9*^low^ glioblastomas. **E** Boxplots showing expression scores for immune exhaustion, macrophages, and TCR/BCR signaling signatures in *SAMD9*^high^ versus *SAMD9*^low^ glioblastomas. **F** Correlation ranking between *SAMD9* expression and immune cell proportions estimated by the xCell algorithm. **G** Representative IHC staining of iNOS, IL-1β, and CD163 in human SAMD9^low^ and SAMD9^high^ glioblastoma specimens. **H** Quantification of IHC staining scores for iNOS, IL-1β, and CD163. Data were presented as mean ± SEM. ***, *p* < 0.001; *, *p* < 0.05; n.s., not significant

Analysis of DEGs between *SAMD9*^low^ and *SAMD9*^high^ Glioblastoma, IDH-wildtype samples from TCGA (|log2FC|> 2, FDR < 0.05) identified 18 significant DEGs, including 15 upregulated and 3 downregulated genes (Fig. 6a). Notably, all 15 upregulated DEGs were ISGs. Significant correlations were observed between *SAMD9* and key ISGs such as *RSAD2*, *MX1*, *OAS3*, and *IFI44L* at bulk transcriptomic level (Fig. S6A), aligning with *SAMD9*’s established role as an IFN-γ response gene [44] and experimentally validated (Fig. S6B). GSEA using Hallmark and Reactome collections further confirmed the enrichment of pathways related to IFN-γ response, IFN-α response, and inflammatory response in *SAMD9*^high^ tumors (Figs. 6b and S7A). To investigate the mechanistic basis for poor treatment response, we examined the interferon-related DNA damage resistance signature (IRDS), a known mediator of radio- and chemotherapy resistance [45]. IRDS was significantly enriched in *SAMD9*^high^ glioblastomas relative to *SAMD9*^low^tumors (Fig. 6c), with similar patterns observed in the CGGA cohort (Fig. S7B-D).

Immune landscape profiling revealed distinct immunological characteristics in *SAMD9*^high^ glioblastomas. These tumors exhibited elevated scores for nearly all immune signatures from Rooney’s collection [46], except for a CD8+ T-cell signature (Fig. 6d). Furthermore, *SAMD9*^high^ tumors consistently showed upregulation of immune exhaustion markers, TAM-related signatures, and TCR/BCR signaling pathways (Fig. 6e). Deconvolution of the TME using *CIBERSORT* and *xCell* algorithm confirmed abundant macrophage infiltration, particularly of inflammatory M1-polarized macrophages, in *SAMD9*^*high*^ tumors (Figs. 6f and S8).

IHC validation demonstrated increased expression of the M1 markers iNOS, IL-1β, as well as the M2 marker CD163, in SAMD9^high^ glioblastoma tissues relative to SAMD9^low^ tissues (Fig. 6g, h). This suggests that SAMD9-high tumors maintain an inflamed yet immunosuppressive microenvironment, which is conducive to immune evasion.

### Prediction of SAMD9-targeting drugs and molecular docking

**Fig. 7:**
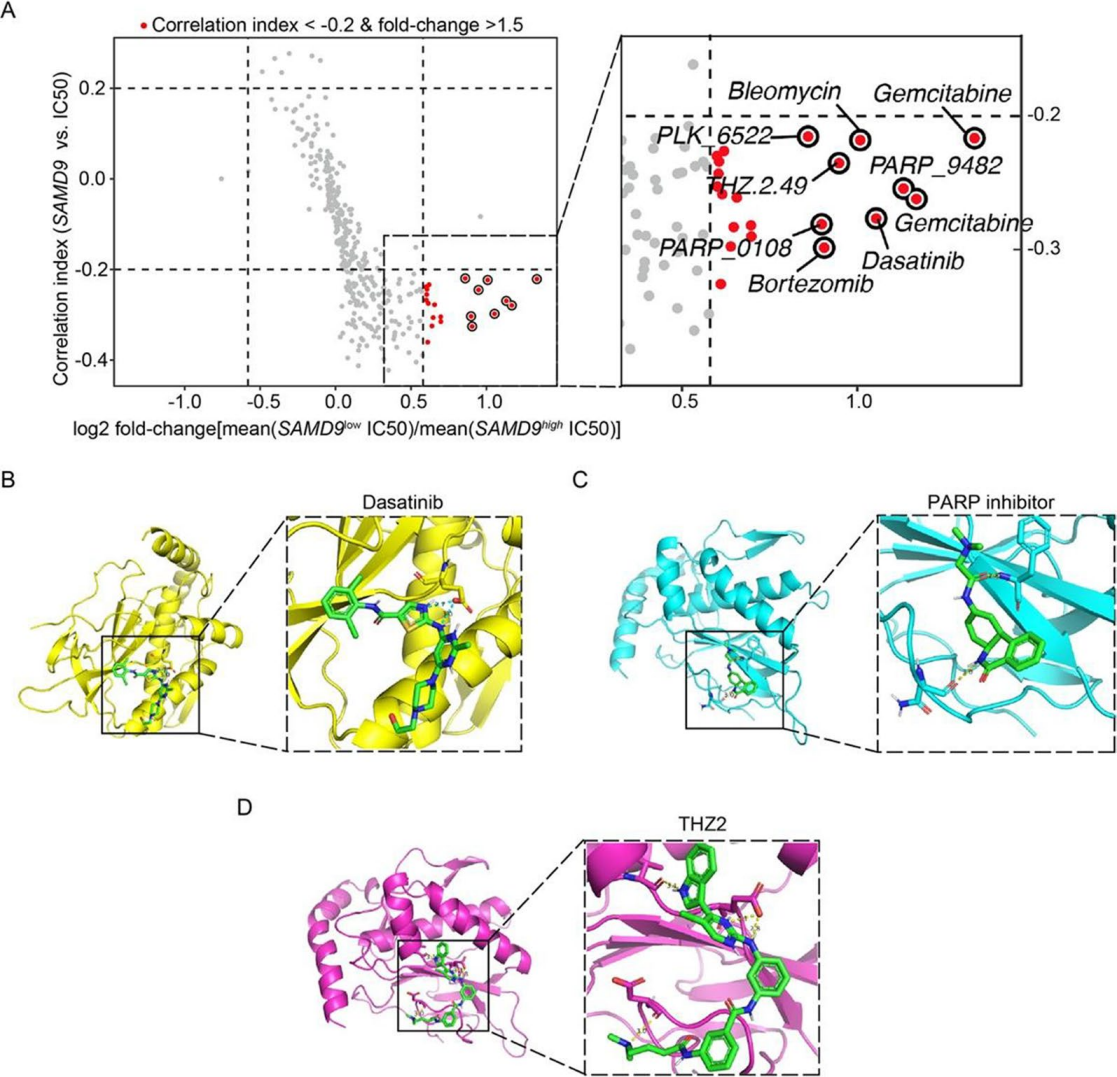
*Prediction of SAMD9-targeting drugs and molecular docking.* **A** Scatter plot of SAMD9-correlated drug candidates. The x-axis represents the ratio of mean IC50 values in *SAMD9*^high^ versus *SAMD9*^low^ glioblastomas, while the y-axis represents the correlation index between the IC50 value and *SAMD9* expression. Drugs meeting with the criterion of correlation index < -0.2 and fold-change > 1.5 are marked in red. **B**–**D** Molecular docking models of SAMD9 protein with Dasatinib **(B)**, PARP inhibitor **(C)**, and THZ2 **(D)**, illustrating binding conformations and intermolecular interactions

To identify potential therapeutics for IDH-wildtype glioblastoma, predicted IC50 values of 365 compounds were correlated with *SAMD9* expression across TCGA samples (Table S4). This analysis identified several candidate drugs, including Bortezomib, Dasatinib, Gemcitabine, Bleomycin, THZ2, a PLK inhibitor, and a PARP inhibitor (Fig. 7a). We subsequently performed molecular docking using Autodock tool to evaluate binding affinity of these candidates to the SAMD9 protein structure (Table S5). Among them, Dasatinib, a multi-target protein tyrosine kinase inhibitor with known efficacy in preclinical glioblastoma models [47], demonstrated strong binding to SAMD9 (Fig. 7b). Dasatinib formed two hydrogen bonds, with a simulated binding energy for molecular docking of − 4.81 (kcal/mol), and the donor-acceptor distances of less than 3.0 Å (Table S5). Intriguingly, Dasatinib treatment downregulated the *SAMD9*-correlated signature (Fig. S9A). Other candidates, such as a PARP inhibitor and THZ2, exhibited even higher binding energies of − 6.03 (kcal/mol) and − 5.65 (kcal/mol), respectively (Fig. 7c, d). They respectively formed two and four hydrogen bonds. However, these compounds failed to significantly downregulate *SAMD9*-correlated signature in glioma cell lines (Fig. S9B, C). Based on its strong binding and functional efficacy, we identified Dasatinib as a promising therapeutic candidate for *SAMD9*^*high*^ glioblastoma.

## Discussion

Glioblastoma, IDH-wildtype, represents the most common and aggressive primary brain tumor, posing significant challenges due to its biological complexity and the difficulty in developing effective therapies. This complexity is compounded by substantial heterogeneity within both the tumor cells and the surrounding microenvironment. In this study, we applied an integrated multi-omics approach combining scRNA-seq, spatial transcriptomics, and extensive bioinformatics resources to systematically characterize the biological functions, immunological features, spatial distribution, and therapeutic vulnerabilities associated with SAMD9 in this disease.

Our identification of SAMD9 as an IFN-γ-responsive protein in glioma aligns with its known roles in antitumor and antiviral activities [13, 44, 48]. While IFN signaling is essential for activating ISGs and orchestrating adaptive immune responses, persistent IFN-γ exposure can induce the upregulation of immune checkpoint molecules such as PD-L1, IDO1, and TIM3, leading to feedback inhibition and therapy resistance [49–51]. Our findings indicate that *SAMD9* is co-expressed with these immune inhibitory molecules (*PD-L1, IDO1*) within immunosuppressive tumor subset, potentially attributed to sustained IFN stimulation. This observation is consistent with the paradoxical role of IFN signaling in immunotherapy. Although high IFN signaling is associated with poor prognosis in glioblastoma [21], it enhances T-cell activation and antitumor immunity in several other malignancies [52]. This context dependency may stem from the opposite functions of enriched IFN signaling in immune cells versus cancer cells that influence the clinical response to ICI [53]. The limitations inherent in bulk expression analysis highlight the necessity of scRNA-seq studies with a large sample size to resolve these issues. Recent work emphasizes the importance of pre-encoded responsiveness to type I IFN in determining anti-PD-1 therapeutic outcomes [19], and the transient efficacy of PD-1 blockade in glioblastoma may reflect peripheral IFN-I reactivity. Further studies are warranted to uncover the detailed mechanism underlying the role of the IFN/SAMD9 axis in reshaping ICI response.

Spatial transcriptomics has emerged as a powerful tool for resolving cellular localization and tissue architecture [37]. Recent advances in computational deconvolution methods allow for a quantitative assessment of cell type composition within each spot [54]. By integrating IvyGAP data and IHC, we discovered predominant SAMD9 expression in peri-necrotic regions, which are typified by pseudopalisading cells, HIF-1α overexpression, and poor clinical outcome [38, 55]. The formation of pseudopalisades is thought to result from tumor cells migrating to the periphery of a central hypoxic field due to vascular occlusion or damage [56]. These migratory, oxygen-deprived cells promote extracellular matrix degradation, VEGF secretion, and microvascular hyperplasia [8]. Our observation of marked *SAMD9* expression in the peri-necrosis region and its upregulation under hypoxia suggests it may facilitate aggressive tumor expansion and pseudopalisade organization. This histologic pattern may be exploited by Bevacizumab, which can exacerbate invasiveness and treatment resistance in preclinical and clinical specimens [41, 57–59]. SAMD9 may thus serve as a predictive biomarker for Bevacizumab response, though mechanistic validation remains necessary. Regional hypoxia drives genomic instability and chromosomal alterations, aiding in the evolution of therapy-resistant clones [5]. The relationship between *EGFR* genomic alteration and SAMD9-positive tumor cells, which are characterized by an immunosuppressive phenotype [21], highlights the need for future work to investigate clarify how *EGFR* genomic changes sculpt microenvironmental evolution and cellular niche formation in glioblastoma. Our investigation into tumor-host interactions revealed that SAMD9-positive  tumor cells are in close proximity to both M1 and M2 TAMs. In SAMD9-high tumors, the strong association between SAMD9 and IFN signaling suggests early involvement of M1-like macrophages. However, sustained activation of the IFN-SAMD9 axis appears to drive TME remodeling toward an immunosuppressive phenotype, characterized by M2-like polarization and immune exclusion—a transition consistent with findings reported by Ma et al. [18]. Thus, the coexistence of M1 and M2 markers likely reflects a dynamic plasticity within the macrophage compartment, rather than a static distribution.

Together, these results imply that SAMD9-high glioblastomas establish an immunosuppressive niche via specialized immune constituencies, adaptive stress responses, and ICI resistance mechanisms. To overcome these barriers, we performed molecular docking-based drug screening and identified Dasatinib, a multi-kinase inhibitor targeting, including BCR-ABL, SRC family kinases, c-KIT, and PDGFR-β [60], as a promising candidate. Preclinical evidence indicates that Dasatinib potently suppresses tumor growth [47, 61] and synergizes with TMZ. However, its clinical translation has been hampered by subtype-specific response variations [62, 63]. Interestingly, *SERPINH1*, a typical *SAMD9*-correlated gene, may help identify patients likely to benefit from Dasatinib [64], supporting the rationale for targeting *SAMD9*-associated signaling. Further research is essential to optimize patient stratification and validate Dasatinib efficacy in *SAMD9*-driven glioblastoma subsets.

## Conclusion

In summary, our study establishes SAMD9 as a central adaptive mediator in IDH-wildtype glioblastoma, linking hypoxic stress and immune suppression to drive tumor progression and therapy resistance. Its expression pattern predicts poor survival following anti-angiogenic therapy and ICI, thereby establishing its dual utility as a stratifying biomarker and a promising therapeutic target. Our drug screening further nominates Dasatinib as a potential strategy for SAMD9-high disease. Future work should focus on delineating the precise mechanisms of SAMD9 and validate this therapeutic candidate in vivo.

## Supplementary Information

Supplementaty Material 1

Supplementaty Material 2

Supplementaty Material 3

Supplementaty Material 4

Supplementaty Material 5

Supplementaty Material 6

## Data Availability

The datasets utilized in this study are accessible from various repositories: GlioVis: http://gliovis.bioinfo.cnio.es/. UCSC Xena: https://xenabrowser.net/. GTEx portal: https://www.gtexportal.org/home/index.html. GEO: https://www.ncbi.nlm.nih.gov/geo/. IvyGAP: http://glioblastoma.alleninstitute.org/static/home. The specific names of the repositories and accession numbers associated with the datasets are provided within the article. Any processed data presented in this study can be obtained upon request from the corresponding author.

## Abbreviations

AC: Astrocyte
ATCC: American Type Culture Collection
CGGA: Chinese Glioma Genome Atlas
CoCl_2_: Cobalt chloride
CPTAC: Clinical Proteomic Tumor Analysis Consortium
CNS: Central Nervous System
DEG: Differential expressed gene
DMEM: Dulbecco’s Modified Eagle Medium
FBS: Fetal bovine serum
G-CIMP: Glioma CpG island methylator phenotype
GDSC: Genomics of Drug Sensitivity in Cancer
GEO: Gene Expression Omnibus
GSEA: Gene set enrichment analysis
GTEx: Genotype-Tissue Expression Project
HGG: High-grade glioma
IC50: Half-maximal inhibitory concentration
ICI: Immune checkpoint inhibitor
IHC: Immunohistochemistry
IFN: Interferon
IRDS: IFN-related DNA damage resistance signature
ISH: In-situ hybridization
ISGs: Interferon-stimulated genes
IvyGAP: Ivy Glioblastoma Atlas Project
LGG: Low-grade glioma
MES: Mesenchymal
MsigDB: Molecular Signatures Database
NPC: Neural progenitor cell
NES: Normalized enrichment score
OPC: Oligodendrocyte progenitor cell
PDB: Protein Data Bank
RBC: Red blood cell
RT: Radiotherapy & Temozolomide
SAMD9: Sterile Alpha Motif Domain Containing 9
scRNA-seq: Single-cell RNA-sequencing
ssGSEA: Single-sample Gene Set Enrichment Score
TAMs: Tumor-associated macrophages
TC: Tumor cell
TCGA: The Cancer Genome Atlas
TME: Tumor microenvironment
TIL: Tumor-infiltrating lymphocyte
TMA: Tissue microarray
TMZ: Temozolomide
WT: Wild-type
