# Supplementary material for: Identification of SAMD9 as an adaptive response gene to environmental changes and its association with overall survival and immunotherapeutic response in glioblastoma
Source: Cancer Cell Int. 2025 Dec 2;26:9. doi: 10.1186/s12935-025-04068-3 (PMC12798092; doi:10.1186/s12935-025-04068-3)
Supplement: Supplementary file 1 — Supplementaty Material 1 [file 12935_2025_4068_MOESM1_ESM.doc]

**Supplymentary Figures**

**
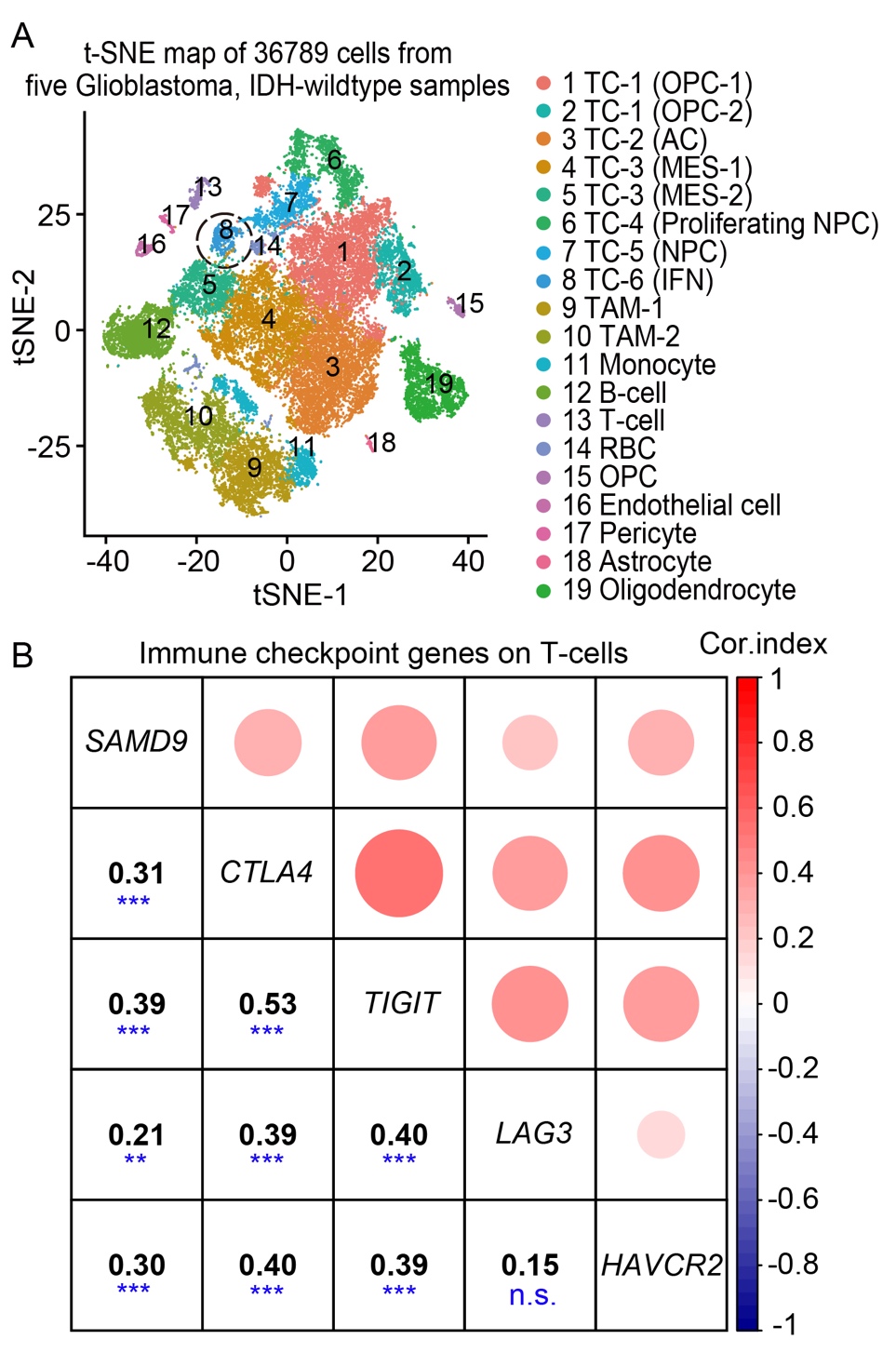
**

**Supplementary Fig. 1** (A) The t-SNE plot illustrating 36789 cells from five **Glioblastoma, IDH-wildtype** samples with cell type annotations as previously defined. The TC-6 (IFN) subset is highlighted. TAM, Tumor-associated macrophages; RBC, Red blood cell; OPC, Oligodendrocyte progenitor cell; NPC, Neural progenitor cell; AC, Astrocyte; MES, Mesenchymal; IFN, Interferon. (B) Correlation analysis between *SAMD9* and T-cell-expressed immune checkpoint genes. In the upper triangular, circle size and color represent the correlation strength gene expression level, while the lower triangle displays Pearson correlation coefficients. ***, *p* < 0.001; **, *p* < 0.01; n.s., not significant.


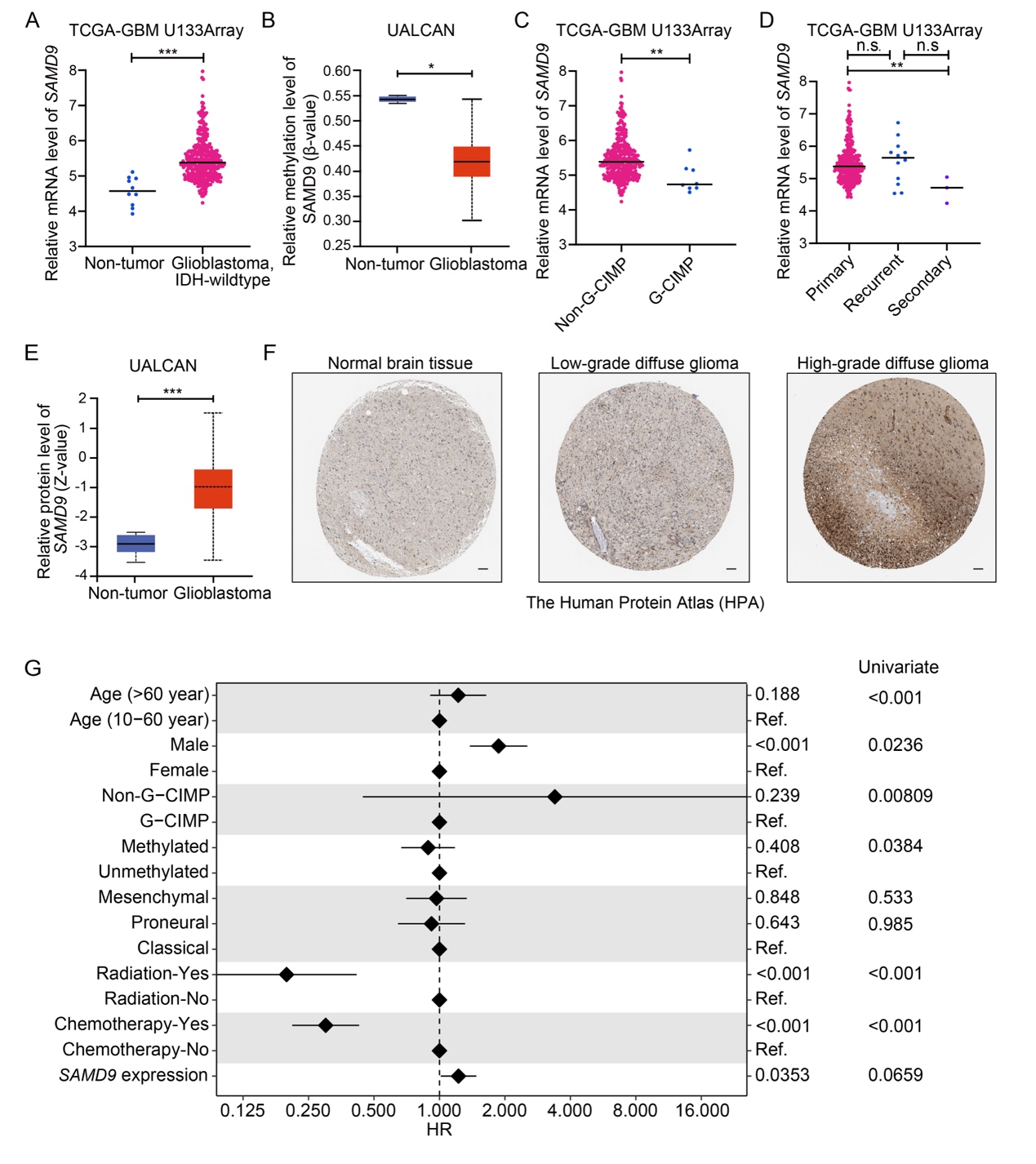


**Supplementary Fig. 2** (A) *SAMD9* mRNA expression level in TCGA-U133 **IDH-wildtype** **glioblastoma** versus normal brain tissue. (B) Histograms of *SAMD9* promoter methylation in normal and **glioblastoma** samples from the UALCAN portal. 0.7＞β-value > 0.5 was considered hyper-methylation, 0.3＞β-value > 0.25 was hypo-methylation. (C-D) Violin plots showing *SAMD9* stratified by Glioma CpG island methylator phenotype (G-CIMP) status (C), and disease stage (primary, recurrent, and secondary) (D) in the TCGA cohort. (E) Histograms of the proteomic level of SAMD9 in normal and **glioblastoma** tissues from the CPTAC portal. (F) Representative immunohistochemistry images of SAMD9 protein expression across glioma grades (Human Protein Atlas). Scale bar = 100 μm. (G) Forest plot of multivariate analysis for IDH-wildtype **glioblastoma** in the TCGA-U133 cohort, showing hazard ratio (HR) and univariate *p*-values. ***, *p* < 0.001; **, *p* < 0.01; *, *p* < 0.05; n.s., not significant.


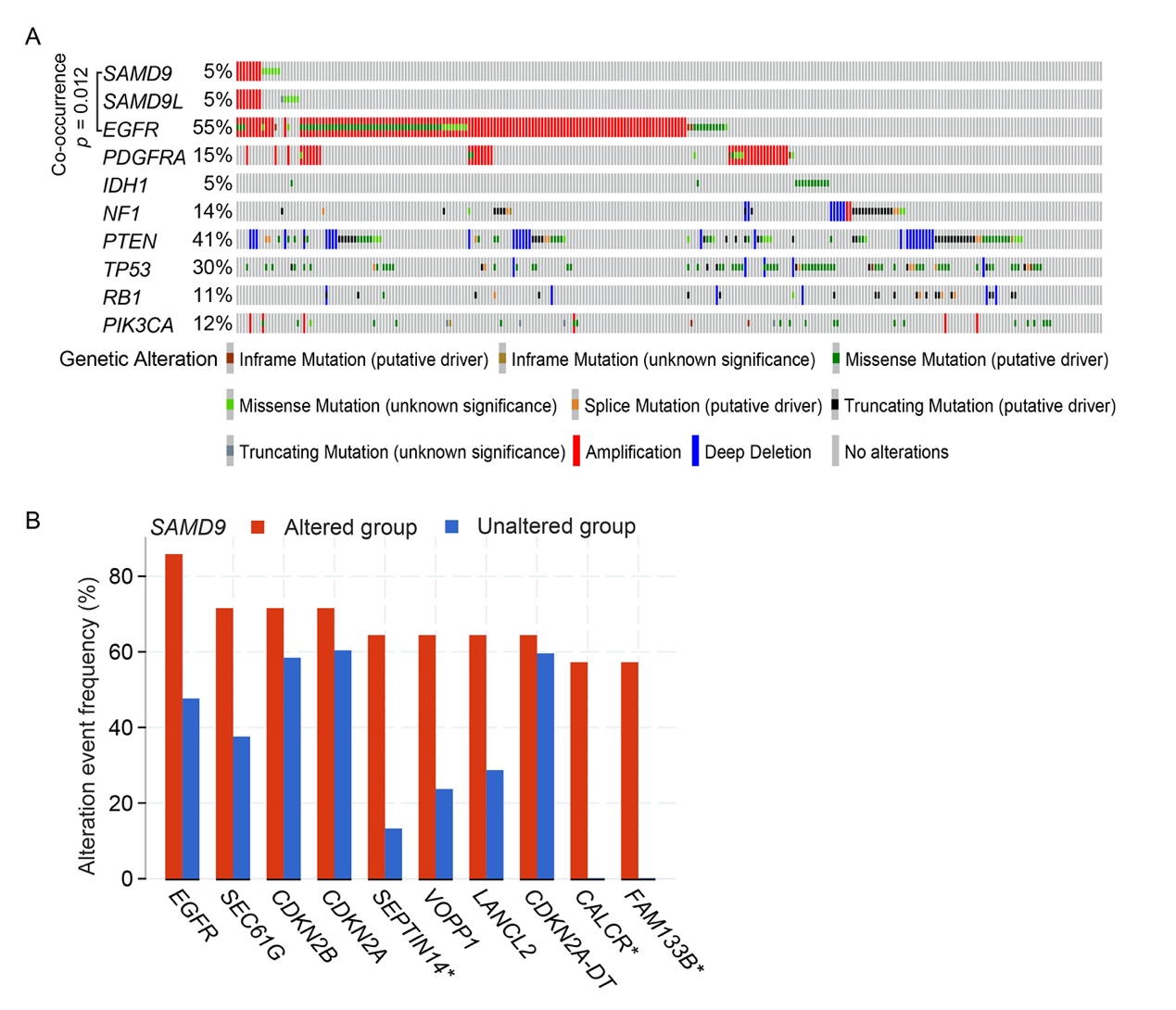


**Supplementary Fig. 3** (A) The Oncoplot depicting genomic alteration frequencies in glioblastoma-related driver genes and *SAMD9/SAMD9L* across the **TCGA glioblastoma** samples. Genes showing statistically significant co-occurrence with *SAMD9* alterations are highlighted. (B) The most frequent alteration events in the *SAMD9* altered group versus the unaltered group. The asterisk (*) indicates significantly occurring alterations.


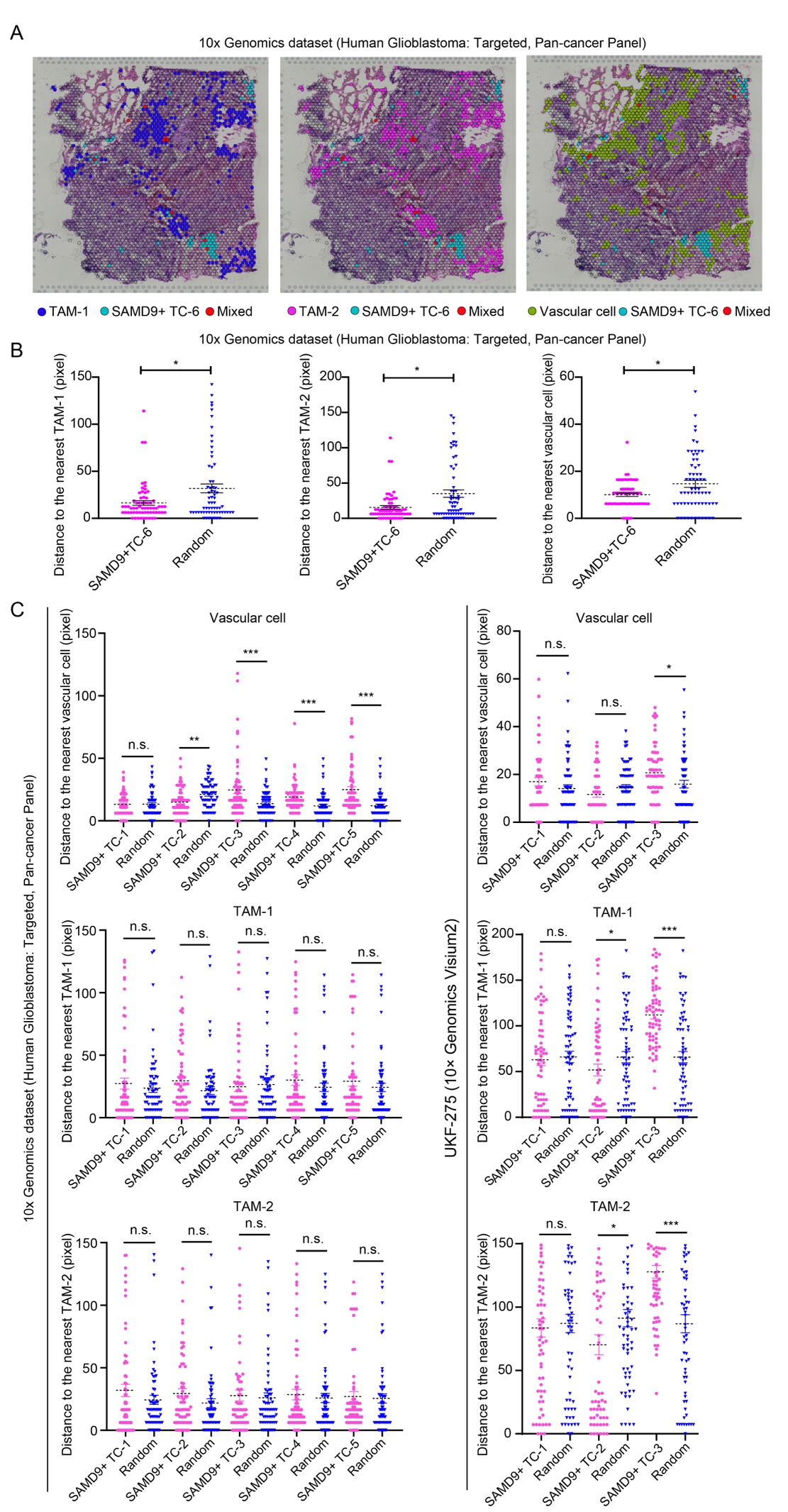


**Supplementary Fig. 4** (A) Spatial mapping of TAMs, vascular cells, and SAMD9-positive tumor subsets on Visium spatial data, with each cell type represented by a different color. (B) Scatter plots showing the distance to the nearest TAM-1, TAM-2, and vascular cells for SAMD9-positive TC-6 cells and their paired random sampling spots. (C-D) Scatter plots illustrating the distance of other tumor cell subsets and their randomly selected sampling spots to the nearest TAM-1, TAM-2, and vascular cells in the 10x Genomics dataset (C) and the UKF-275 sample (D). Of note, TC-4 and TC-5 subsets were not detected in the UKF-275 spatial alignment. ***, *p* < 0.001; **, *p* < 0.01; *, *p* < 0.05. n.s., not significant.


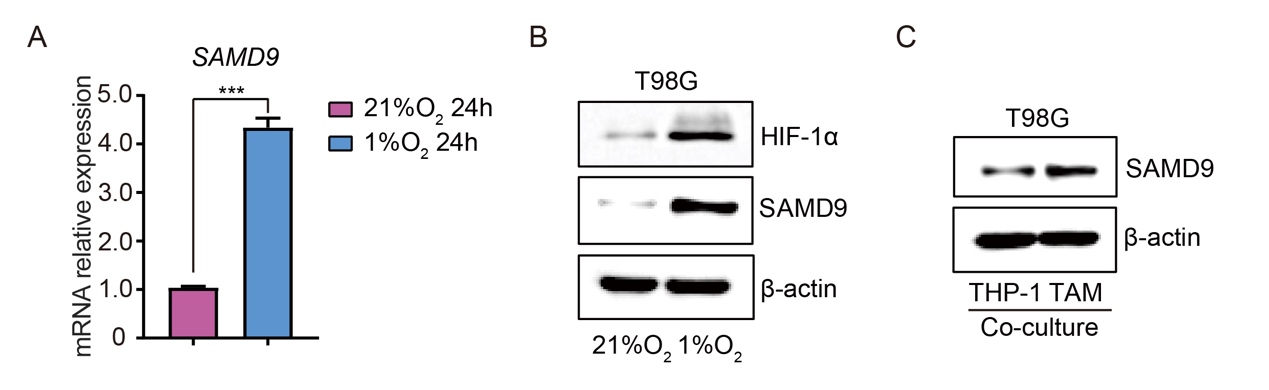


**Supplementary Fig. 5** (A-B) mRNA (A) and protein (B) expressions of SAMD9 were measured at 24 hours after treating T98G cells under hypoxic conditions (1% O_2_). ***, *p* < 0.001. (C) SAMD9 protein expression in T98G cells after 24-hour co-culture with untreated THP-1 monocytes or THP-1-derived TAMs.


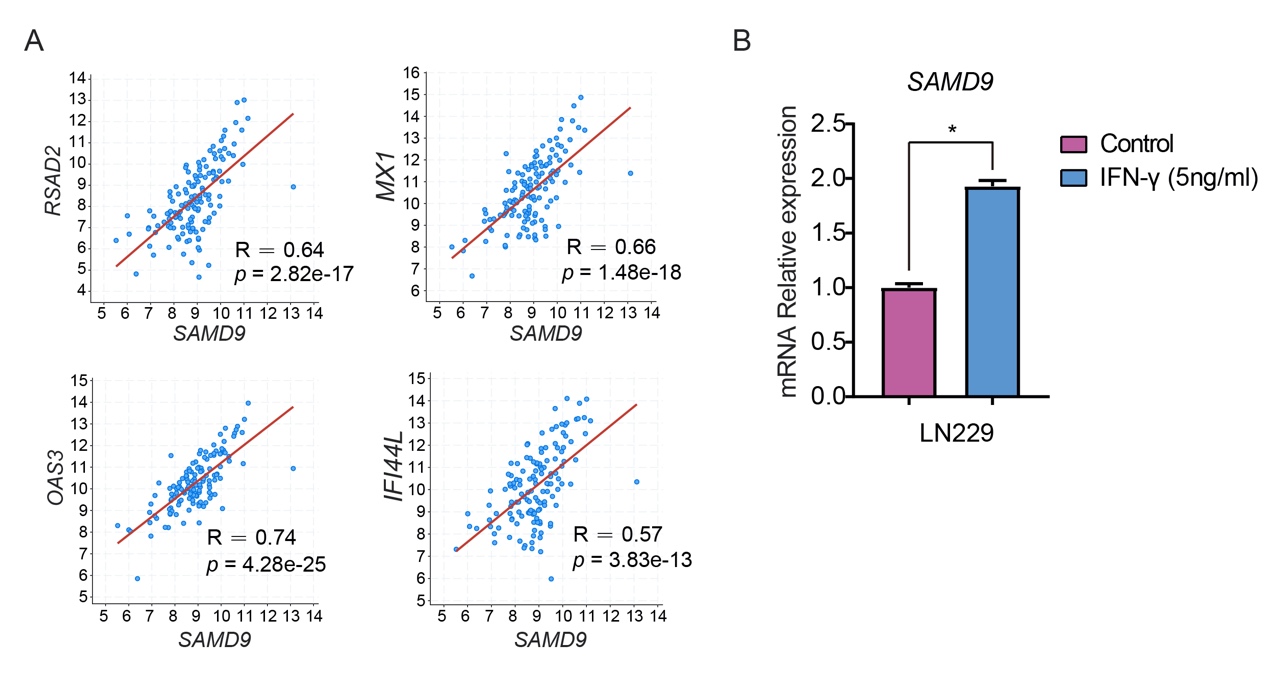


**Supplementary Fig. 6** (A) Correlation analysis between *SAMD*9 and representative interferon-stimulated genes (ISGs) expressions in the TCGA cohort. Pearson correlation coefficients (R) and statistical significance are indicated. (B) *SAMD9* mRNA expression in cells treated with IFN-γ (5 ng/mL) for 24 hours. *, *p* < 0.05.


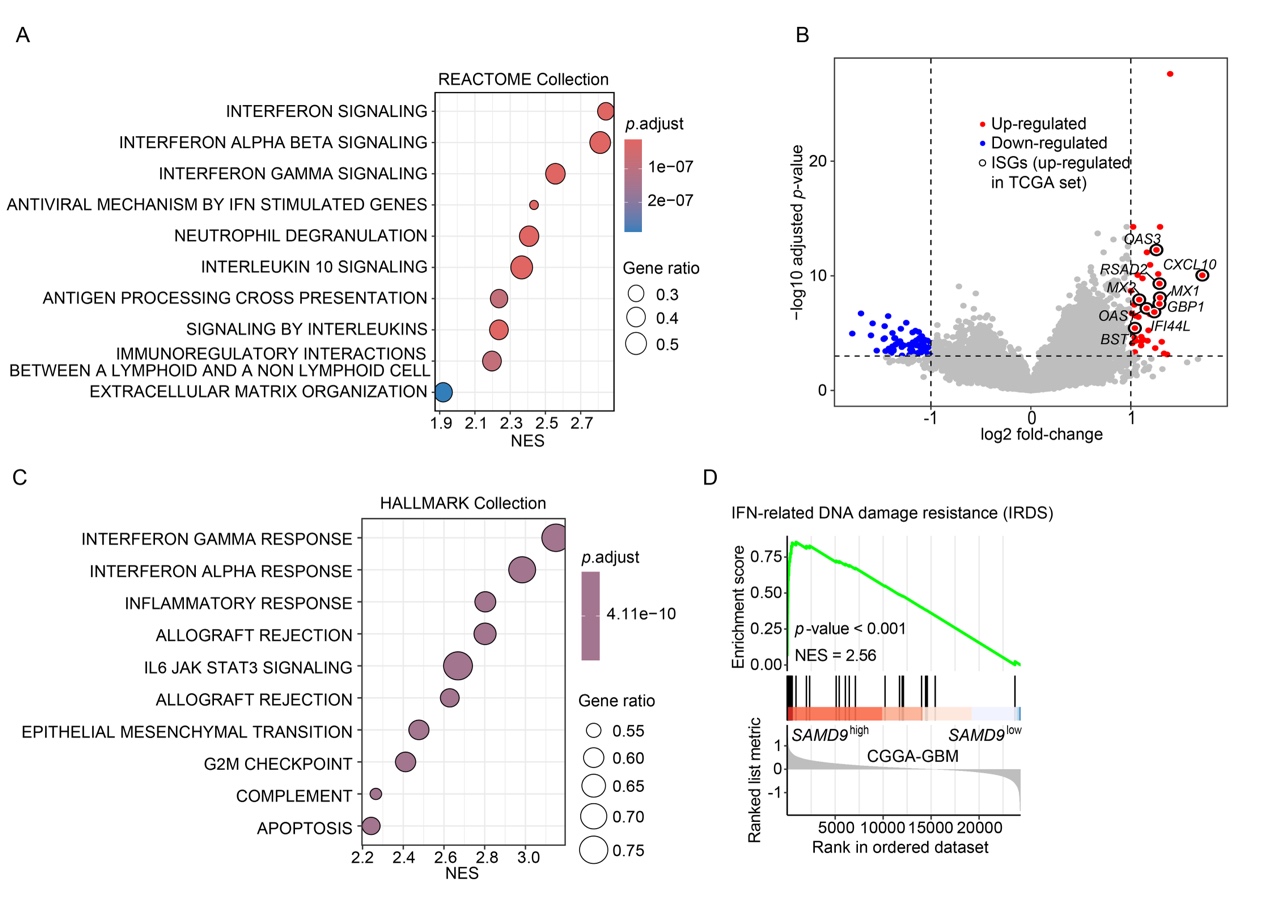


**Supplementary Fig. 7** (A) Dot plots showing gene set enrichment analysis (GSEA) result of differential expression genes (DEGs) in *SAMD9*^high^ versus *SAMD9*^low^ **glioblastomas** in the TCGA cohort. The x-axis shows the normalized enrichment score (NES) for Reactome pathway terms; dot color and size correspond to adjusted *p*-value and gene ratio, respectively. (B) Volcano plot of DEGs in *SAMD9*^high^ versus *SAMD9*^low^ glioblastomas from the CGGA cohort (|fold-change| > 2, *p*-value < 0.05). Upregulated and downregulated genes are marked in red and blue, respectively. Interferon-stimulated genes (ISGs) that were upregulated in the TCGA dataset are circled in black. (C) Dot plot of GSEA for DEGs in *SAMD9*^high^ versus *SAMD9*^low^ glioblastomas from the CGGA cohort. The x-axis shows the NES for Hallmark gene sets; dot color and size correspond to adjusted *p*-value and gene ratio, respectively. (D) GSEA plot demonstrating significant enrichment of the interferon-related DNA damage resistance signature (IRDS) in *SAMD9*^high^ compared to *SAMD9*^low^ glioblastomas in the CGGA cohort.


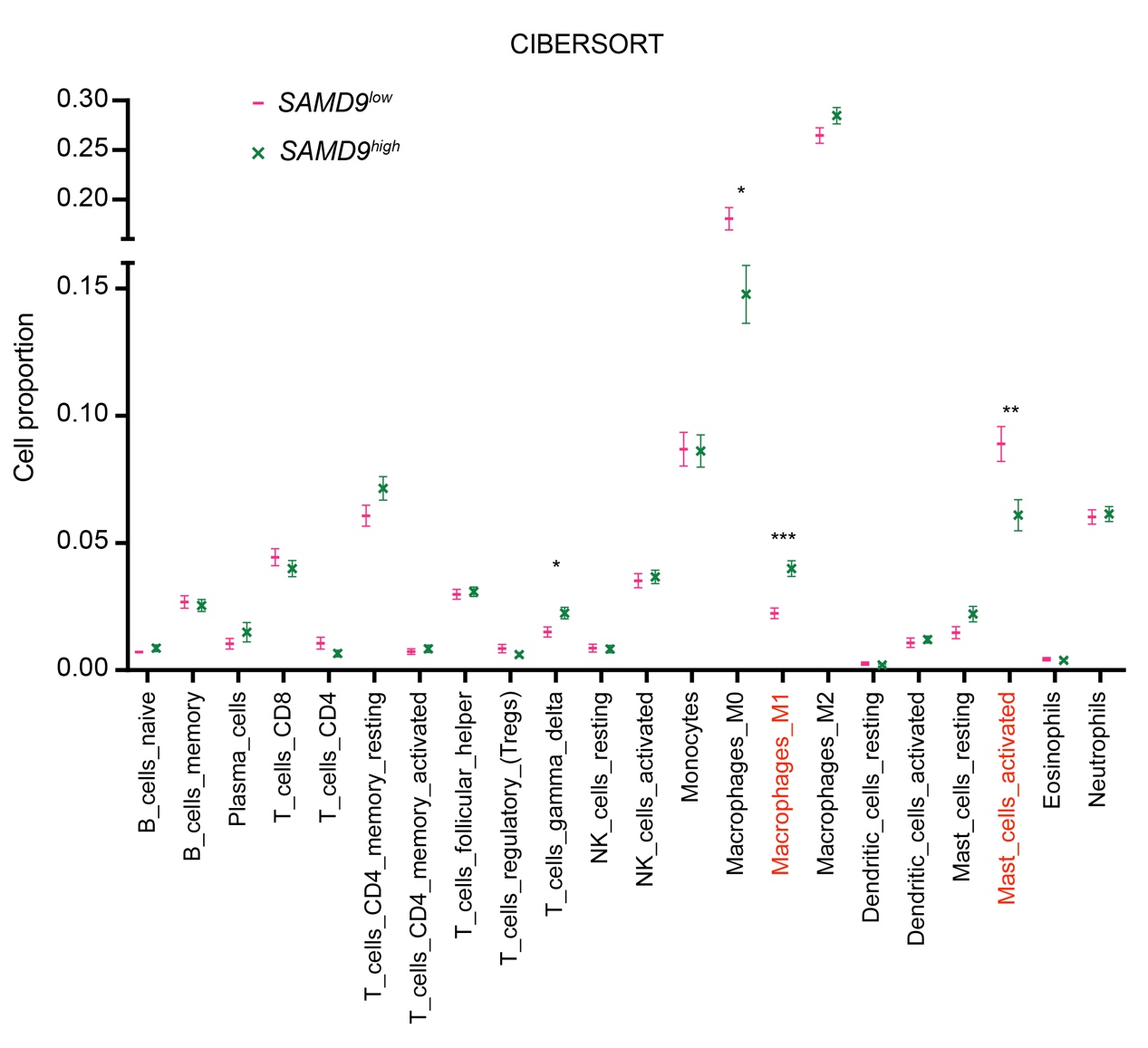


**Supplementary Fig. 8** Comparative analysis of immune cell infiltration in *SAMD9*^high^ and *SAMD9*^low^ glioblastomas. Bar plot showing differences in the estimated proportions of 22 immune cell types between *SAMD9*^high^ and *SAMD9*^low^ glioblastomas, as calculated by the CIBERSORT algorithm. For each cell type, the mean value for each group is plotted, and error bars indicate the SEM. ***, *p* < 0.001; **, *p* < 0.01; *, *p* < 0.05. Non-significant comparisons are not shown.


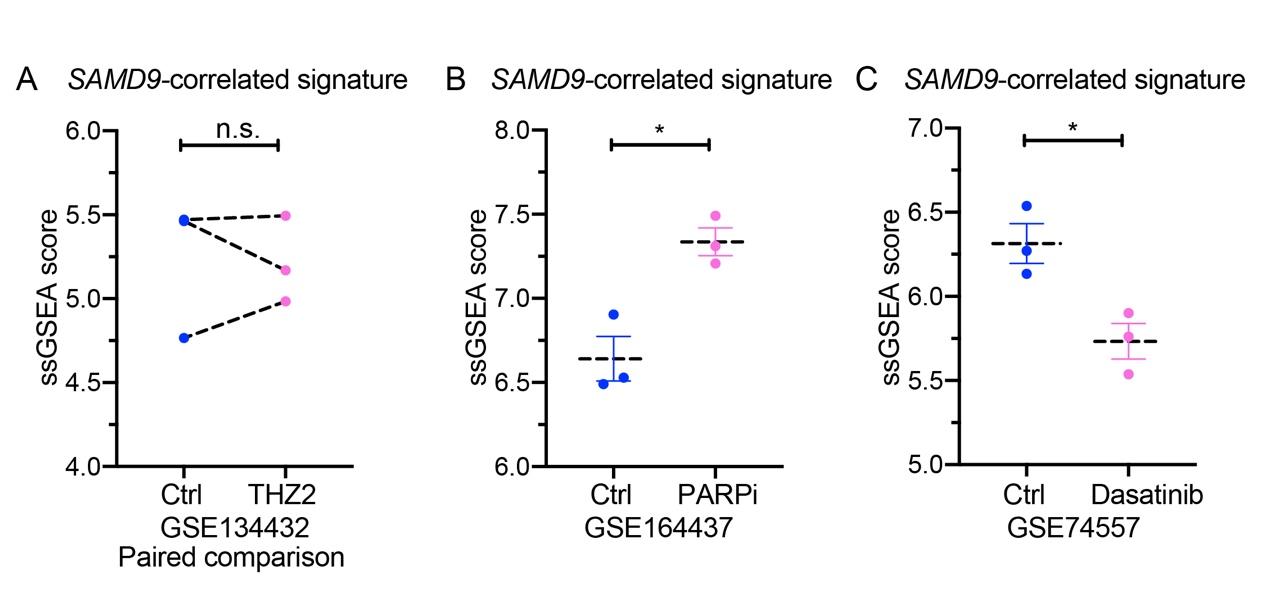


**Supplementary Fig. 9** Single-sample GSEA (ssGSEA) score of the *SAMD9*-correlated signature in control group versus THZ2-treated (**A**), PARP inhibitor (PARPi)-treated (**B**), and Dasatinib-treated (**C**) from microarray datasets. In panels **B** and **C**, horizontal dotted lines and error bars within the scatter plots represent the group mean ± SEM. In panel **A**, dotted lines connect paired samples. *, *p* < 0.05. n.s., not significant.
